# Speckle Tracking Echocardiography for the Assessment of the Athlete’s Heart: Is It Ready for Daily Practice?

**DOI:** 10.1007/s11936-018-0677-0

**Published:** 2018-08-27

**Authors:** Lynsey Forsythe, Keith George, David Oxborough

**Affiliations:** Research Institute for Sport and Exercise Sciences, Liverpool John Moores University, Tom Reilly Building, Liverpool, L3 3AF UK

**Keywords:** Echocardiography, Strain, Athletes heart, Cardiac screening

## Abstract

**Purpose of review:**

To describe the use of speckle tracking echocardiography (STE) in the biventricular assessment of athletes’ heart (AH). Can STE aid differential diagnosis during pre-participation cardiac screening (PCS) of athletes?

**Recent findings:**

Data from recent patient, population and athlete studies suggest potential discriminatory value of STE, alongside standard echocardiographic measurements, in the early detection of clinically relevant systolic dysfunction. STE can also contribute to subsequent prognosis and risk stratification.

**Summary:**

Despite some heterogeneity in STE data in athletes, left ventricular global longitudinal strain (GLS) and right ventricular longitudinal strain (RV ɛ) indices can add to differential diagnostic protocols in PCS. STE should be used in addition to standard echocardiographic tools and be conducted by an experienced operator with significant knowledge of the AH. Other indices, including left ventricular circumferential strain and twist, may provide insight, but further research in clinical and athletic populations is warranted. This review also raises the potential role for STE measures performed during exercise as well as in serial follow-up as a method to improve diagnostic yield.

## Introduction

Echocardiography has advanced our knowledge and understanding of the structural and functional adaptation that occurs in the athletes’ heart (AH) in response to chronic training [1–4]. Whilst many different factors can influence cardiac adaptation in the athlete including sex, age, ethnicity, body size, genetics, training volume, type of sport and competitive level [5], common phenotypic descriptions of the AH have been reported in the literature. Normal physiological remodelling of the left ventricle (LV) can include cavity dilatation, increased wall thickness, normal or slightly reduced systolic function [6] and normal or enhanced diastolic function [1, 3, 7]. Physiological adaptation in the right ventricle (RV) can be characterised by cavity dilatation with normal wall thickness and normal systolic and diastolic function [8, 9].

Cardiac chamber enlargement and impaired functional parameters can be also associated with pathological disease, more specifically, cardiomyopathy including hypertrophic cardiomyopathy (HCM), dilated cardiomyopathy (DCM) and arrhythmogenic right ventricular cardiomyopathy (ARVC) [10]. This creates a diagnostic dilemma or ‘grey zone’ between normal physiological adaptation and potential pathological disease [10]. Although sudden cardiac death (SCD) in athletes is rare (approximately 1 in 50,000) [11], athletes are at greater risk if they have an undetected, underlying condition. In view of this, there is now a growing awareness for the need for pre-participation cardiac screening (PCS) for sports participation to identify those at risk of SCD and it is mandated by many national and international sports federations [12]. Echocardiography is an integral part of PCS, and recent advances in technology such as speckle tracking echocardiography (STE) provide additional global and regional functional assessment of chamber mechanics [13]. This review focusses on the use of strain (ɛ) imaging by STE in the assessment of the AH and assesses the potential of the technique to aid differential diagnosis during PCS.

## Speckle tracking echocardiography—the rationale

Currently, the most commonly used ɛ imaging modality for assessment of cardiac mechanics is STE. This technique allows for estimation of myocardial ɛ to identify local shortening, thickening and lengthening of the myocardium and provides quantitative measurements of LV regional and global function [14]. ɛ describes the myocardial deformation or fractional change in length of a myocardial segment and is expressed as a percentage of its length at end diastole [13]. It can be measured in the longitudinal, circumferential and radial planes of LV motion as well as in the assessment of LV rotation and twist [13].

RV function assessment by conventional 2D echocardiography is challenging due to the complex RV geometry and the heavily trabeculated inner wall contour [15]. Due to the dominance of longitudinal and oblique myocardial fibres in the RV free wall [15], STE can aid functional assessment by the measurement of RV longitudinal ɛ (RV ɛ) [16]. Peak RV ɛ is expressed as a mean of the basal, mid and apical segmental ɛ with normal RV ɛ parameters showing a base to apex gradient with highest values observed at the apex [17].

Standard echocardiographic global functional assessment of the LV is limited by the perception that normal LV ejection fraction (LVEF) equals normal systolic function and that abnormal LVEF equals abnormal function. This may not always be the case and is highlighted by recent studies of pathological hypertension in patients diagnosed with heart failure with normal ejection fraction (HFNEF). HFNEF patients have by definition normal LVEF were systolic function and contractility have been assumed to be normal [18] but there is a paradox of reduced longitudinal, circumferential and radial ɛ with normal absolute radial thickening and therefore LVEF. Normal LVEF can be explained by the increased diastolic wall thickness [19, 20]. The terms LVEF and LV function are not synonymous and in the context of increased wall thickness, normal absolute radial thickening results in normal EF with the illusion of normal pump function [20]. Longitudinal function appears to precede radial dysfunction in many pathological models [21] which has contributed to the adoption of ɛ imaging in the early detection of sub-clinical LV dysfunction as well as being a prognostic indicator [22, 23, 24]. With altered ɛ indices being reported in cardiomyopathy patients [14], the use of STE in cardiac disease highlights the possibility of STE improving the diagnostic capability of echocardiography in the AH. When we consider that many athletes have marked structural remodelling and given that functional abnormalities are likely to be subtle at an early stage, this review raises the question as to whether the addition of STE improves the sensitivity and specificity of echocardiography in PCS?

## Speckle tracking echocardiography and the AH

### LV global longitudinal strain

LV global longitudinal strain (GLS) is the most frequently reported deformation parameter in clinical and AH studies [25••] and it is now considered a more sensitive measure of systolic function than LVEF in the identification of sub-clinical LV dysfunction [14]. Recent meta-analyses and systematic reviews have highlighted a number of STE studies in athletes in comparison to controls, athletes of different sports and patients with cardiac disease [26•, 27•]. The findings are heterogeneous with some studies demonstrating higher GLS in athletes compared to controls [28, 29] others showing no differences [30, 31, 32] and others showing lower values in the athlete [33, 34]. This disparity is likely a consequence of a variation in LV structure secondary to the training type and volume. Variable ɛ parameters were found when assessing different sporting disciplines which were subsequently normalised following indexing for LV end diastolic volume [35]. In addition, a recent publication demonstrates a clear relationship between LV morphology, strain and ejection fraction [36]. Female athletes have also been found to have higher GLS than male athletes [37] suggesting that further research is needed in STE and gender differences. In a longitudinal training study of athletes (soccer, basketball and volleyball) involved in an 18-week training study, only a mild increase in GLS, associated with heart rate (HR) and LV size, was observed despite significant increases in LV mass, LV internal dimension in diastole (LVIDd) and systolic volume [38]. Recent European guidelines [25••] demonstrate that GLS in the general population can be variable, as in athletes, and the current normal GLS range has been reported as − 16 to − 22% with a mean of − 20%.

**Fig. 1:**
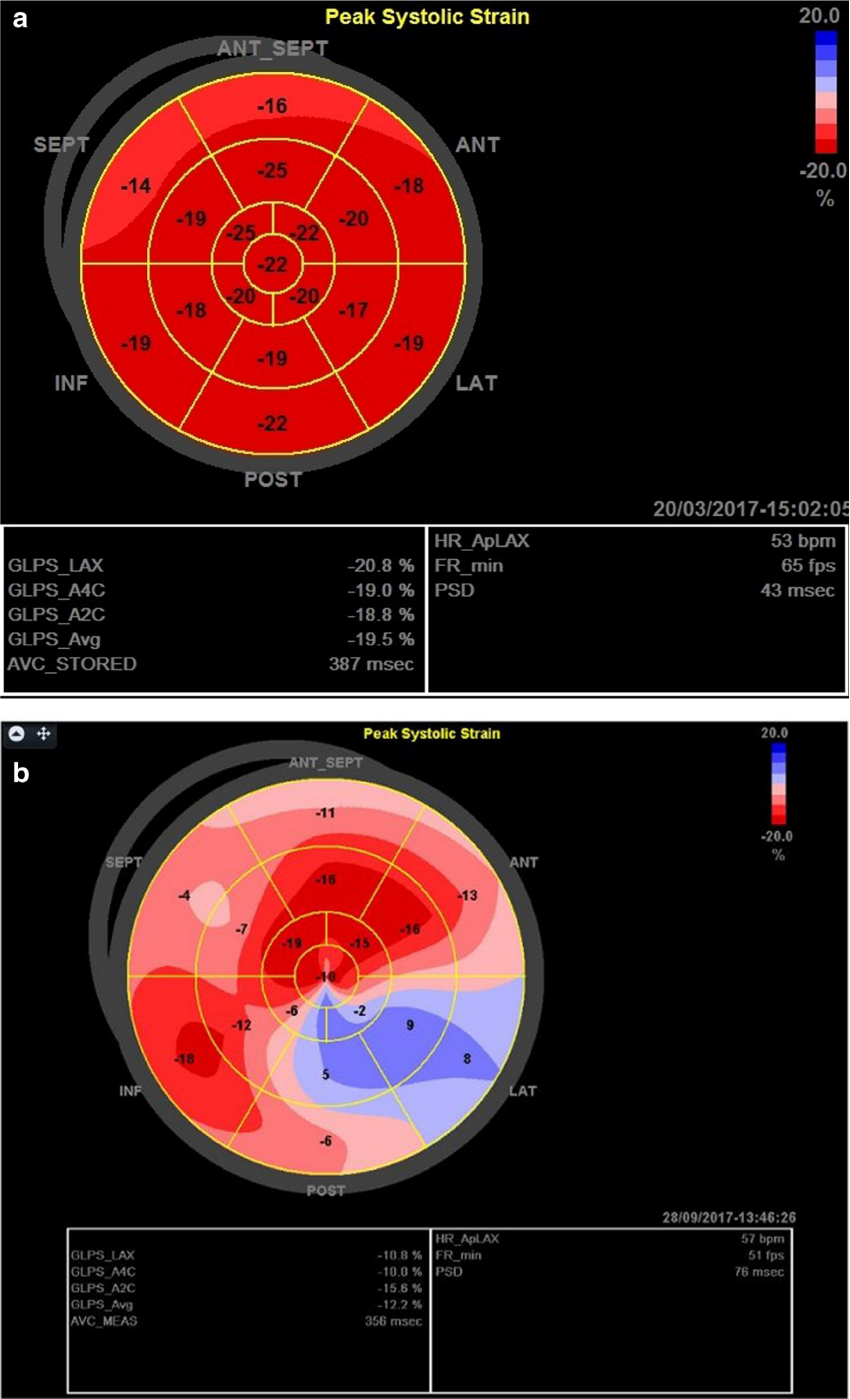
Bullseye representation of GLS. **a** Normal elite athlete. **b** HCM patient.

When considering the differentiation from pathology, a study of athletes, controls and hypertensive patients [39] demonstrated significantly lower GLS in hypertensive patients with another study demonstrating similar findings [40]. These studies highlight the potential of STE and a reduction in GLS as an early sign of LV dysfunction. Patients with HCM have been found to have lower GLS compared to controls [41] with Kansal et al. [33] and Butz et al. [42] demonstrating lower GLS in HCM patients compared to athletes. An exemplar representation of LV GLS in an athlete and an HCM patient is presented in Fig. 1a, b. In a study of DCM patients, decreased GLS was observed even in the setting of normal EF highlighting the potential of GLS as an early marker of DCM and in serial assessment of systolic function [43]. The reduction in GLS in hypertensive, DCM and HCM patients has prognostic significance suggesting a maladaptive association with cardiovascular pathology and therefore offers potential in the differentiation of these conditions from AH. STE may therefore improve the sensitivity and specificity for the differentiation of cardiomyopathy identifying subtle structural-functional alterations. Based on this, the European guidelines suggest that GLS of less than − 15% may be indicative of myocardial disease [25••]. The likelihood of pathology is raised when a low GLS is seen alongside increases in relative wall thickness and/or significant LV dilatation [27•, 33].

### LV circumferential and radial strain

Fewer studies report global circumferential strain (GCS) and global radial strain (GRS) in the AH. Nottin et al. [44] reported little or no differences in circumferential ɛ but observed lower peak apical radial ɛ during systole in cyclists compared to controls. No differences in GCS or GRS were reported in a study of sedentary and trained subjects [45], and likewise, no differences in GCS and GRS were reported in athletes from different sporting categories (endurance, strength, mixed) compared to controls [28]. Szauder et al. [46] reported that bodybuilders have lower GCS than marathon runners which is also supported by data from Utomi et al. [30]. The recent meta-analysis [26•] concluded that there are no differences in GCS between athletes and controls, but when categorised for sporting type, resistance athletes may have lower values creating a diagnostic quandary in those athletes.

GCS and GRS were similar between controls, rowers and early hypertensive patients [40]. Patients with HCM have been found to have lower values of LV radial ɛ and increased circumferential ɛ compared to controls [41]. In a study of STE between soccer players, HCM patients and controls [47], whilst radial ɛ was significantly higher in athletes than controls, compared to HCM patients, athletes had higher values of radial and circumferential ɛ [47], and therefore a disproportionate shift in mechanics may provide additional differential utility.

### Regional LV STE

Few studies have reported comprehensive data sets for regional STE data across longitudinal, circumferential and radial planes [36, 48] but heterogeneity in ɛ has been reported in all planes and is likely related to physiological structural remodelling. Knowledge of normal physiological regional adaptation may add additional relevant information when investigating abnormal global parameters. Reporting of regional ɛ in all cardiac planes of motion in athletes involved in a range of sporting disciplines would provide valuable information in this regard.

### LV twist

LV twist from helically orientated fibres is a key component of myocardial performance and can be determined by STE. LV twist data from STE is concordant with torsion measurements from tagged MRI studies in patients with a variety of cardiac pathologies [49]. In a number of studies, twist and apical rotation has been found to be lower in endurance (cyclists) compared to strength (weightlifters) athletes and controls despite any difference in longitudinal ɛ [50]. Similarly, twist and apical rotation were lower in rugby football league (RFL) athletes compared to controls and associated with an increase in basal rotation and no change in longitudinal ɛ [36]. Also, in RFL athletes, lower twist and apical rotation were observed in native Hawaiian and Pacific Island athletes compared to Caucasian counterparts, suggesting that there may also be significant ethnic differences [51]. Lower apical rotation and twist has been observed in amateur swimmers of different ages (16–48 years) with higher longitudinal ɛ compared to controls [52]. Similarly, lower values of twist have reported in soccer players [53] and cyclists compared to controls [44]. The reduction in twist appears to be predominantly driven by reduced apical rotation, and it has been reported that the LV apex may be more dependent on sympathetic activity than the LV base [44]. This equivocal finding may be related to training induced sympathovagal balance and could be interpreted as a functional reserve to aid oxygen and substrate delivery to the muscle during exercise [27•]. In contrast, higher twist has been exhibited in endurance (marathon runners) and mixed trained (martial arts) athletes compared to controls [28] and further evidence of higher twist in resistance athletes compared to controls was reported by Beaumont et al. [26•]. Whilst the evidence suggests that twist appears to be lower in endurance athletes, some data would suggest that an increase in twist in resistance athletes is a normal phenomenon. There are few longitudinal studies assessing LV twist but there is evidence of a phasic phenomenon in twist parameters. In rowers, following 3 months of training twist was higher than baseline but after 39 months, twist was lower suggesting both acute and chronic exercise effects on this parameter [54].

Higher LV twist has been reported in hypertensive patients compared to controls with no difference between athletes and controls [39], but in contrast, twist has been also found to be similar in athletes, controls and newly diagnosed hypertensive patients [40]. An increase in twist with pathology and a preserved EF could be an early indicator of systolic dysfunction as the LV compensates for a reduction in longitudinal function in pathological left ventricular hypertrophy (LVH) [55]. No differences in twist, apical or basal rotation parameters were observed in a study between athletes, HCM patients and controls [56]. Despite this, peak twist occurred after aortic valve closure exclusively in HCM patients suggesting that a late, lower and slower untwist may be able to differentiate from pathology. In contrast, untwist was higher in elite athletes occurring earlier and faster. Untwist and untwist rate correlated with E/A ratio and the early diastolic phase was the most discernible component of the cardiac cycle [56]. Similarly, Pacileo et al. [57] have demonstrated prolonged LV twist in cardiomyopathies. These studies indicate the potential clinical benefit of twist and untwist in differential diagnosis, not only by the use of peak values but also through a temporal assessment.

### Right ventricular longitudinal strain

Resting RV ɛ parameters in endurance athletes [8] have been reported to fall within the reported normal population range (− 18 to − 39%) [58]. There are reports of a higher global RV ɛ in top level rowers compared to sedentary controls [59]. Conversely, lower RV ɛ has been reported in elite endurance athletes compared to controls, specifically in those athletes with an associated dilated RV cavity, largely due to lower basal strain [60]. This finding was reproduced in a subsequent study but with the additional finding of an increase in apical segment strain [61]. The authors proposed that normal physiological adaptation in the AH resulted in a base to apex gradient in RV deformation [60, 61], and whilst this gradient was also observed by Utomi et al. [62], no regional differences in RV ɛ were found in this study. Some studies also report no difference in RV ɛ between endurance and strength-based athletes [62, 63]. Lower RV ɛ has been observed in endurance (marathon runners), strength (power lifters) and mixed trained athletes (Martial arts) with increases observed with exercise [28]. In a seasonal study, despite increases in RV size in basketball and volleyball players during the season, RV function and global RV ɛ did not change. There were some regional changes with RV apical ɛ increasing from pre-season to end season [64]. It is clear that RV ɛ in the AH is variable and may well be related to RV enlargement/geometry [65]. Global RV ɛ rarely falls outside of normal range but the regional changes at the apex and base may compound the diagnostic differentiation particularly as regional abnormalities have been identified in asymptomatic patients who are carriers of genetic mutations for ARVC [66].

Peak systolic RV ɛ is significantly reduced in ARVC patients compared to controls and RV STE has been used to identify regional wall abnormalities in patients [66] making STE superior to conventional echocardiography in identifying the disease [66]. Compared to the LV, there are limited RV STE studies in athletes and there is a lack of universally accepted cut-off values [25••]. A recent and large meta-analysis of RV structure and function in ARVC [67] concluded that RV ɛ is significantly lower in ARVC patients compared to controls with a range of RV ɛ in controls of − 27 to − 31% and in ARVC patients of − 13 to − 21%. A cut-off for pathology of less than − 21% was therefore suggested.

### Should an exercise stimulus be considered during assessment of STE in AH?

HCM patients have been found to have lower LV longitudinal and radial ɛ at rest with higher circumferential ɛ and twist compared to controls. Exercise has induced a modest increase in longitudinal ɛ in HCM patients but not in twist whilst both parameters increased in controls [41]. In pre- and post-exercise studies between athletes (soccer players) and controls using hand grip exercise, EF was not different but mid to apical longitudinal ɛ although similar at rest, was higher in athletes post-exercise. The authors suggested that exercise produces enhanced ɛ in the mid to apical segments and may represent a regional functional reserve [68]. There is some data to suggest that an exercise stimulus is useful for differentiating DCM and AH, as identified with LVEF [69]; however, this has not been applied to ɛ imaging and therefore, future work should aim to establish the exercise response in the differentiation of these conditions.

La Gerche et al. [61] reported a normal physiological response of the RV to exercise, likely due to an enhanced cardiac reserve of the basal segment. Functional athletic adaptation may not be apparent at rest and it was proposed on this basis that stress echocardiography may aid differential diagnosis in the RV [61]. Findings of lower segmental RV ɛ have led to concern over possible detrimental effects of chronic exercise on RV structure and function [61, 70]. Following an ultramarathon, the RV has been found to be significantly increased in size with a decrease in RV ɛ post-race with a concomitant reduction in LV ɛ. This was described by the authors as cardiac fatigue from which athletes later recovered; however, it is not yet clear what long-term impact repeated bouts of post-exercise dysfunction will have [71]. In a similar study, endurance athletes were studied at baseline, immediately post-race and 1 week post-race with decreased RV function, including RV ɛ post-race but mostly a complete recovery was observed after 1 week [70]. Short- and long-term effects of exercise on RV function in athletes of different discipline require further investigation.

As reduced RV ɛ can be an early marker of disease in ARVC [14, 72, 73], the assessment of ɛ, as an early indicator for RV dysfunction [13], may be useful in the differential diagnosis of AH or pathology in PCS. Further work on RV STE parameters in athletes is required, but it is a promising technique to quantify regional RV dysfunction [66] and the elucidation of normal RV STE parameters (regional, global and temporal) at rest and during exercise has the potential to promote the clinical application of the technique. ARVC is not only a disease of the RV, as it can also have an impact on LV myocardial tissue [74]. Establishing the extent of LV involvement in ARVC may be useful in diagnostic and/or prognostic decision making.

### Is STE ready for daily practice in the assessment of AH?

A major limitation of the clinical application of STE is the lack of universally accepted STE data collection protocols and normative data for healthy adults, athletes and cardiac patients. Variability in specific data can be related to vendor-to-vendor differences [75], software version and upgrades [14, 76] and skill of the operators performing STE analysis [22].

Despite some technical limitations, we suggest on current evidence that LV GLS and RV ɛ should now be included in the standard echocardiographic assessment of AH. We are not suggesting that STE parameters are used solely for differentiation of physiology and pathology in AH, but used together with standard structural and functional echocardiographic parameters they may aid in the differentiation of AH from HCM, DCM and ARVC. STE has the potential to reveal early cardiac dysfunction and may corroborate conventional findings, increasing sensitivity and specificity of PCS. There is an increasing evidence base for normative GCS, GRS, regional and twist parameters in athletes, and whilst the potential of these parameters has been eluded to in this review, it is our opinion that much more work is needed before these parameters can be routinely used in AH assessment.

In conclusion, clinicians should be familiar with the assessment of AH and STE analysis should be performed by experienced operators. If STE is applied with caution and with an understanding of the limitations, global and regional ɛ, LV GLS and RV ɛ will provide additional information to aid the differential diagnosis of physiological and pathological adaptation.
